# Article upon “Cruelty to Animals” Reviewed

**Published:** 1867-04

**Authors:** 


### Article upon "Cruelty to Animals" Reviewed.

It appears that our Clerical and other friends, thought rather poorly of our article upon the subject of *cruelty to animals*, "that it was unfriendly and false," and it was to be regretted that a "medical man standing so high as Dr. Miner should write or be responsible for such an article." We are very much obliged for the complimentary manner in which we are abused, and no complaint is going to be made about it.

Mr. Fillmore, we believe, speaks of a letter received from Mr. Bergh previous to the movements in Buffalo. The first announcement of a public meeting here, stated that it would be addressed by an agent of the New York Society. We submit if these two facts did not justify our saying that we thought the movement was "in secret sympathy with the New York Society." This sympathy is denied, and our statements characterized as false. We acknowledge the error, and pray to be forgiven for inserting the word *secret;* when that is omitted, the truth of it is evident.

To the charge of being unfriendly, we propose to plead guilty, for we have an inward conviction of unfriendliness, which had grown out of no unkindness to the ladies and gentlemen of Buffalo, who had interested themselves in this movement. We should dislike to be outdone in politeness, and will here assure the Officers and members of the Society of our hearty approval of, and general interest in, the legitimate objects of their association, which if conducted with judgment and care, may be the means of effecting great good. They are ladies and gentleman of culture and scientific attainment, and any interest may be safely entrusted to their keeping; even *vivisection* for the purposes of scientific advancement. We were confessing to some unfriendliness, but were diverted a little, to return the compliments received, and only desire briefly to intimate some of the reasons for our opposition. Mr. Bergh, President of the N. Y. Society, had rendered himself most unspeakably disagreeable to us, by his offensive letters, by his exaggerated and untruthful public descriptions of physiological experiments, by his offensive interference in the provisions of the State act, to prevent cruelty to animals, and by his whole course in administering the affairs of that Society. We believed that Buffalo had gained some inspirations from this, in many respects ignorant, and otherwise than in these efforts, idle reformer, and that if the affairs of the Buffalo Society were to fall into similar keeping, it would prove a similar nuisance. Some of the remarks in the public meetings, as published in the daily papers upon the subject of vivisection, appear related to Mr. Bergh's offensive and untruthful representations; but all sympathy has been denied, and we have no doubt the affairs of the Society here, will be conducted in such manner as to meet general approval; still we think it better not to deny many of the statements made in our article. We regret want of space, to place before our readers the whole facts and history of this movement; we are in possession of documents which would instruct many of those who are engaged in it, and show them why the movement has been ridiculed and opposed. An enterprise which commends itself to the liberal and humane, has been made odious and disgusting by a presumptuous willingness in those who conducted it, to undertake reforms, in matters of which they were profoundly ignorant.

